# Vehicle Detection in Overhead Satellite Images Using a One-Stage Object Detection Model

**DOI:** 10.3390/s20226485

**Published:** 2020-11-13

**Authors:** Delia-Georgiana Stuparu, Radu-Ioan Ciobanu, Ciprian Dobre

**Affiliations:** 1 Faculty of Automatic Control and Computers, University Politehnica of Bucharest, RO-060042 Bucharest, Romania; delia.stuparu@stud.acs.upb.ro (D.-G.S.); ciprian.dobre@upb.ro (C.D.); 2 National Institute for Research and Development in Informatics, RO-011455 Bucharest, Romania

**Keywords:** object detection model, satellite images, vehicle detection, smart city

## Abstract

In order to improve the traffic in large cities and to avoid congestion, advanced methods of detecting and predicting vehicle behaviour are needed. Such methods require complex information regarding the number of vehicles on the roads, their positions, directions, etc. One way to obtain this information is by analyzing overhead images collected by satellites or drones, and extracting information from them through intelligent machine learning models. Thus, in this paper we propose and present a one-stage object detection model for finding vehicles in satellite images using the RetinaNet architecture and the Cars Overhead With Context dataset. By analyzing the results obtained by the proposed model, we show that it has a very good vehicle detection accuracy and a very low detection time, which shows that it can be employed to successfully extract data from real-time satellite or drone data.

## 1. Introduction

An overview of the Earth from above can offer a plethora of information regarding the anthropogenic impact of the technological evolution from the last decades. Satellite images offer such data, which can be extremely useful in cartography, meteorology or monitoring. At the same time, traffic flow at the ground level is one of the main global issues, especially in urban areas. Monitoring traffic in a uniform manner is an important challenge in the present day. The solution used until recently is to employ surveillance cameras and sensors. However, there is a need for traffic monitoring on a large scale so that images obtained from video cameras can be validated by overview images, without the need for human intervention.

In this context, many methods of vehicle detection have been proposed in recent years. The largest part of such approaches is dedicated to first-person perspectives, where the sought-after object has a large size. These models are generally applicable on video-captured images, offering information about spatially-restricted areas.

However, these images do not offer an overview, so, if we are talking about real-time decisions such as traffic light synchronization, they often prove useless. In such cases, overhead images might be the solution. Furthermore, these kinds of images offer data useful for creating statistics and even making predictions regarding traffic dynamics in the long term. In addition, collecting such images can be performed in a more localized and cheaper fashion, through the use of IoT-based solutions such as unmanned aerial vehicles (UAVs) [1], which would be able to fly above the roads of a neighborhood or a city and collect overhead images (either for later use or for live detection of vehicles and traffic monitoring). In order to improve the capabilities of such drone-based networks, they can be complemented with device-to-device communication in an opportunistic fashion [2], which would reduce the need for a large cloud or server infrastructure when collecting, aggregating and processing the data, since this could be done at a more local level.

A model trained to detect cars from overhead images with a high precision will be able to operate on drone-collected data, which would offer a major boost in today’s environment. Thus, creating such a model is the first step towards real-time vehicle detection. For these reasons, this paper has two major goals:

- identify vehicles in satellite images with a high precision
- create a model that is capable to detect vehicles from aerial images (such as drone-captured pictures) in real-time.

The necessity of not only identifying vehicles in satellite images, but doing so in a real-time fashion, is given by the fact that, in static images, the state of the detected vehicles cannot be easily inferred. The cars can be parked, they can move, they can have a certain direction, etc. Therefore, additional information is required, which is why such a detection needs to be performed continuously on live images captured by drones or sensors, thus forming the backbone of an intelligent traffic decongestion system. Such a system can even be extended by connecting the vehicles themselves to the infrastructure and making them act as sensors, consequently increasing the amount of available context knowledge.

For an even more complex analysis of traffic data, vehicle detection can be augmented with street detection [3], since the areas where the vehicles should be sought would be reduced to road-covered sections of the map. Thus, detecting roads and vehicles can have a very good applicability in smart cities, where real-time traffic monitoring and scheduling solutions are required.

For these reasons, in this article we propose and present a model for detecting vehicles in satellite images, using the RetinaNet architecture [4] and the Cars Overhead With Context dataset [5]. This model is able to detect vehicles in overhead images collected by both satellites and drones. We focus on two important aspects of such a model, the first one being the detection precision. Thus, we present a gradual approach, showing how our model evolved through various improvements, finally leading to good results that are presented later on. Furthermore, we are also interested in detecting vehicles in real-time, so another important metric that we analyze and highlight is the detection time, which shows that our model can be successfully applied to real-time monitoring.

The rest of this paper is structured as follows. In Section 2, we present related work in the area of vehicle detection and, more specifically, detecting cars in satellite images. Then, we describe the solution in a detailed fashion in Section 3. Next, we present our implementation in Section 4 and show the results we obtained in Section 5. Finally, we highlight our conclusions and present future work in Section 6.

## 2. Related Work

### 2.1. Vehicle Detection in Satellite Imagery

In contrast to first-person images, aerial views have several shortcomings [6] regarding the following aspects:

- the objects have small dimensions (15–30 pixels for a car).
- the position of an object is not fixed (i.e., it can be rotated).
- the image size might reach hundreds of megapixels.
- the number of available datasets is limited.

On the other hand, real distances and the size of the image are always known, so it is easy to compute dimensions. Furthermore, the observation angle is constant.

classic approach for satellite-based object detection involves a sliding window and a classifier that decides if, in that specific area, the sought object is present or not. For this, the image is processed using techniques like Canny edge detection [7] and Histogram of Oriented Gradients (HOG). The sliding window method can exhibit good results without a high computational effort and with no need for GPU processing. However, because there are overlaps between the sliding windows, some objects may be counted multiple times. The solution is to keep only the highest score detection, using Non-Maximum Suppression [8]. We have also applied this algorithm in the current research.

Two-stage detectors, such as Faster R-CNN [9], have encountered a significant success in object recognition. The first step is to identify the areas of interest and the second one is to apply classification and regression only over those zones. The Satellite Imagery Multiscale Rapid Detection with Windowed Networks (SIMRDWN) Architecture [6] implements the functionality of the two-stage detector You Only Look Twice (YOLT) [10], using the Tensorflow Object Detection API (https://github.com/tensorflow/models/tree/master/research/object_detection). It solves the shortcomings of a similar one-stage algorithm, You Only Look Once (YOLO) (https://pjreddie.com/darknet/yolo/), whose main limitation is the incapacity to detect crowded groups of objects or objects with different scales.

Even if this method fits the aim of detecting cars in aerial images, it comes with the trade-off of long detection times. One-stage detectors are faster because they do not contain the first step (i.e., selecting the area of interest), but directly apply classification and regression. In this category, YOLO and Single Shot Detector (SSD) [11] have promising results, but their accuracy is still low for small and dense objects.

Other novel and interesting neural network-based solutions for object detection in aerial images are proposed and presented in [12,13], but the purpose is somewhat more general than the goal of our paper, since the authors focus on multiple classes of aerial images and not particularly on vehicles. Moreover, the datasets employed by the authors do not include the much more detailed Cars Overhead With Context dataset that we employ here.

Ki et al. perform a survey on the existing datasets for aerial image detection, and propose their own benchmark for remote object detection, entitled DIOR [14]. This dataset can be employed for multiple use cases, since it contains 20 object classes. Although we did not utilize it for this paper (choosing instead to focus on a vehicle detection-oriented dataset that we considered more suitable for showcasing our solution), we plan to analyze and use the DIOR benchmark in the future.

### 2.2. The RetinaNet Architecture for Object Detection

The RetinaNet architecture is an innovation that combines the performances of two-stage detectors with the rapidity of one-stage methods. A specific use case of this network is to identify lesions in Computed Tomography (CT) [15]. In the current research, we used some anchor configurations (as shown in Section 3.2.1) used in CT research, in order to locate small objects.

Regarding vehicle detection in satellite imagery, some remarkable results have been obtained during multiple competitions. In 2019, a solution that uses RetinaNet won the third place in the Esri Data Science Challenge (https://www.hackerearth.com/challenges/hiring/esri-data-science-challenge-2019/). Their model was able to detect cars and pools in 224 × 224 px aerial images, obtaining a mean average precision score of 0.7709.

In 2018, the winning team of the NATO Innovation Challenge got remarkable results regarding vehicle detection in aerial images. According to their article [16], they obtained an F1-score (metric explained in Section 5.1) of 0.95. In their research, they applied a RetinaNet network on the same dataset employed in the current study. However, their main problem was that the model considered ventilation shafts on top of buildings to be cars [16], which is the reason why the F1-score had low values for some areas with easy to confuse objects.

We considered their approach as being the starting point in our novel research. For our proposed solution to exhibit better results, our goal was to improve the detection accuracy and precision by adding negative examples during training. This way, we were able to eliminate some confusion caused by those objects that were similar to the vehicles that needed to be detected, by labelling them as “non-cars”. The implementation of our solution was a gradual process that consisted of training the network with various parameters and adding additional optimizations at each step, as described in Section 4 and Section 5.

## 3. Proposed Solution

Our solution combines the complexity of the RetinaNet architecture with the generality of the Cars Overhead With Context dataset (COWC). The main challenge was to process the images from the dataset, such that the training results are satisfying, regardless of the analysis data. We also had to find the best training parameters for the network and to run it multiple times.

### 3.1. The COWC Dataset

The image type plays an essential role in the way of training the RetinaNet network. After a thorough analysis of the available datasets, we found out that the COWC dataset is one of the few sets that could be used for our purpose. Datasets like ImageNet (http://www.image-net.org/), PASCAL VOC (http://host.robots.ox.ac.uk/pascal/VOC/) or MS COCO (http://cocodataset.org/##home) do not contain aerial images, but the pre-computed weights could be used as a starting point for fine-tuning. In this paper, we started from the weights computed for the MS COCO dataset and we recalculated them for the COWC images. Other popular sets with aerial images are VEDAI [17], COWC [5], DLR-MVDA [18] and KIT AIS (http://www.ipf.kit.edu/downloads_data_set_AIS_vehicle_tracking.php), presented in Table 1.

The VEDAI dataset [17] is frequently used in the literature, but it only contains images from the AGRC Utah collection, so it does not cover a wide variety of geographic areas. Instead, Cars Overhead with Context (COWC) [5] contains 32,716 unique cars, in pictures from six distinct areas: Toronto (Canada), Selwyn (New Zealand), Potsdam and Vaihingen (Germany), Columbus and Utah (USA). All the images are taken from aerial platforms, and their resolution of 15 cm/px enables us to apply detection algorithms over them.

In the car class, we consider all vehicles and vans, whereas trucks and big cars are not annotated. We have also labeled negative examples in the non-car class. Here, we place objects that might easily be confused with cars, such as boats, bushes or ventilation shafts.

According to its official documentation [5], the COWC dataset has three main objectives. The first one is the classification of objects into the car or non-car classes. It was extended to car type recognition, annotated in the auxiliary dataset, COWC-m (ftp://gdo152.ucllnl.org/cowc-m/datasets/). The second objective is close to the purpose of our research, namely to detect, with high precision, the cars in images. The last one is to estimate the density of vehicles in order to predict traffic.

For our solution, we split the dataset into training and testing sets, considering multiple criteria. First of all, we eliminated the images from Columbus and Vaihingen because they were black and white, and the RetinaNet architecture works with three-colour channels. We also kept all the images from Utah for testing, because of their high number of vehicles and their complexity of context. During the training process, we used images from Toronto (Canada), Selwyn (New Zealand) and Potsdam (Germany).

In the initial dataset, the vehicles were marked with a red point in their centre, and the negative examples with a blue point. We created a script that, based on the centre point, sets the coordinates of the corners. For this, we considered a car having three meters in length, which corresponds to a 20 × 20 px box, for the 15 cm/px resolution image.

The size of ground truth images was an impediment in passing them directly to the network. Regarding the training images, those from Potsdam have 2220 × 2220 px and those from Toronto 11,500 × 7500 px. The pictures from Selwyn are even larger, with a 18,075 × 18,400 px size. The testing images, from Utah, have sizes ranging between 5878 × 5878 px and 13,333 × 13,333 px.

Because of the high values of dimensions, it was hard to train a model able to recognize cars in all situations. Therefore, our solution was to develop a sliding window that cuts the original images into 1000 × 1000 px patches, with a 200 px overlap. The obtained images were then rescaled and sent to the network for training.

### 3.2. The RetinaNet Architecture

As an improvement to the already existing models described in Section 2.2, RetinaNet comes with two significant changes. In terms of structure, the benefit comes from using a Feature Pyramid Network [20]. Furthermore, the cross entropy (https://ml-cheatsheet.readthedocs.io/en/latest/loss_functions.html) function is replaced by the focal loss [4] metric, as shown in Equation (1), for much better results.

(1) $$FL\left(p_{t}\right)=-{\left(1-p_{t}\right)}^{\gamma }log\left(p_{t}\right).$$

In the focal loss formula, we used **$p_{t}$** to define the probability that an object (in this case, a vehicle) is correctly classified:

(2) $$p_{t}=\{\begin{array}{c} p,\;correct\\ 1-p,\;wrong \end{array}$$

In contrast to the cross entropy, the logarithmic function from Equation (1) is multiplied by ${\left(1-p_{t}\right)}^{\gamma }$, while the modulation factor γ oscillates between 0 and 5. For correctly classified objects, $p_{t}$ has a high value, so the calculated value for focal loss is small. It means that the error for gradients is small, so the network should not modify the weights by considering this example. The network has to learn from examples with a large value of error, and then to reduce the error accordingly.

**Figure 1:**
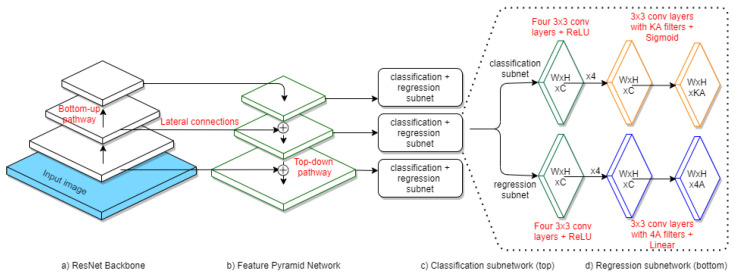
RetinaNet architecture [4]. The bottom-up pathway is a feedforward ResNet Architecture (**a**). The Feature Pyramid Network (FPN) (**b**) is the backbone network for RetinaNet and it is build using lateral and top-down connections. Each pyramid level presents two subnetworks: the classification subnetwork (**c**) and the regression subnetwork (**d**). Convolutional layers are applied on each feature map, having specific characteristics: W—width; H—height; C—channels (256); A—anchors (9), K—classes (2).

Regarding the structure, the RetinaNet architecture contains a base network called a Feature Pyramid Network and two specific sub-networks, for classification and regression, described in this section. The complete architecture of the network can be observed in Figure 1.

#### 3.2.1. Feature Pyramid Network

The base network is a Feature Pyramid Network (FPN) [20] built on a ResNet-50 backbone [21]. This FPN is a convolutional network that has three types of connections (lateral, bottom-up, and top-down), in order to build the convolutional feature map for each input image.

The outputs of the ResNet-50 backbone are the bottom-up connections. The pyramidal network is built using top-down and lateral links. It contains levels from P3 to P7, each one having a resolution $2^{p}$ times lower than the input, where *p* is the level index. The P3, P4 and P5 levels are built on the outputs on ResNet, using top-up and lateral connections. P6 is obtained by applying the 3 × 3 convolution over the previous level’s output, and the P7 level is built using the ReLU (rectified linear input) activation function. The P2 level was omitted because the resulting images were too large and the computational effort would have been significant (especially when the goal is to obtain real-time analysis, as is the case in our solution).

In order to detect multiple objects in a picture, we used reference frames called anchor boxes. Each pyramid level contains anchors of specific sizes, from $32^{2}$ for P3 to $512^{2}$ for P7 [4]. Furthermore, each anchor has three dimension ratios (1:2, 1:1, 2:1) and three scales ($2^{0}$, $2^{\frac{1}{3}}$ and $2^{\frac{2}{3}}$), resulting in a total of nine types of anchors per level.

We trained the network using default anchor dimensions, but the results were not satisfactory enough, so we decided to optimize them based on the conclusions presented in [15]. This way, we obtained optimal values for dimension ratios (0.653, 1.0, 1.531) and for scale (0.404, 0.5, 0.647).

To overlap an anchor box with a specific bounding box, we used *Intersection over Union* (https://www.pyimagesearch.com/intersection-over-union), which specifies that, if the score is over 0.5, it means that the object is located inside the anchor box. A score between 0.4 and 0.5 shows that the network should not learn from this example, because the presence of the object is ambiguous. Finally, a score below 0.4 means that the anchor does not contain a relevant object, but only background information [22].

#### 3.2.2. The Classification Subnet

The classification subnet is a *Fully Convolutional Network*, attached to each level of the FPN, as shown in Figure 1c. It contains four 3 × 3 convolutional layers, a ReLU activation function, followed by another 3 × 3 convolutional layer, and finally the Sigmoid function. Its goal is to predict the object’s existence in a specific position in the image.

#### 3.2.3. The Regression Subnet

The regression subnet, also shown in Figure 1, is a Fully Convolutional Network that predicts the position of an object by computing the offset between the anchor and the real object. The network’s design is identical with the classification subnet, with the single difference being the final activation function which, in this case, is the linear one [4].

## 4. Implementation

For the implementation, we first had to preprocess the images from the dataset, then to prepare the training environment with the appropriate parameters, and finally to perform an evaluation of the obtained model.

### 4.1. Dataset Preparation

As presented in Section 3.1, the Cars Overhead With Context dataset contains images that are too large to be passed directly through the network. The position of the car is represented by the coordinates of its centre point. To train the model, we had to crop the original images in smaller patches and to determine the coordinates for the framing rectangles of the cars.

**Figure 2:**
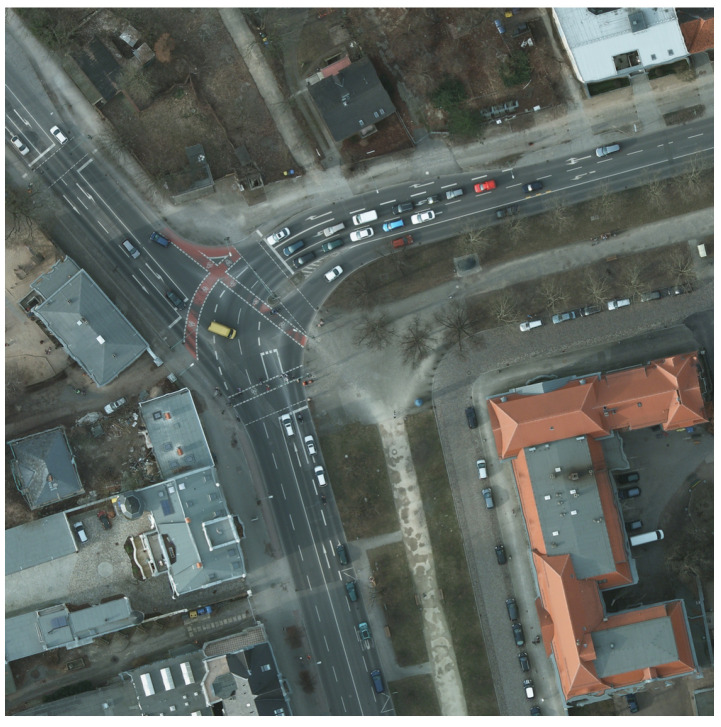
Sample image from the training set (“top_potsdam_5_10_RGB_800_800.png”).

For cropping, we made a sliding window that cuts the image in 1000 × 1000 px slices, with a 200 px overlap. Figure 2 is a sample extracted from a large original image, and all the following examples will be applied on this slice. Note that the last two values in the filename represent the coordinates of the upper left corner.

**Figure 3:**
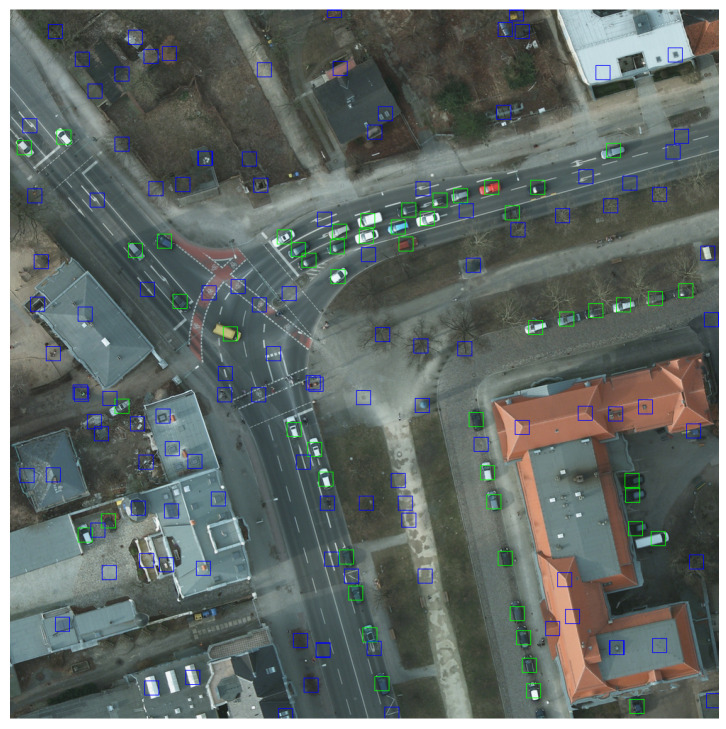
Image obtained after annotating cars (green) and non-cars (blue).

After cropping the original images, we had to determine the coordinates of the cars in all the patches. We generated the bounding boxes for both cars and non-cars, and we considered them having 20 × 20 px. In Figure 3, the cars are framed by green rectangles and non-cars with blue ones.

The information for all the images is written in a CSV file that will be given to the network. For each bounding box, there is a new line in the file containing the path to the image and the coordinates of the car/non-car. There are also images with no significant information, but we kept them into the training process by adding an empty line in the CSV.

### 4.2. Training the Model

For training the model, we used the open-source implementation of RetinaNet, built with Keras 2.3.0 and Tensorflow 2.1.0. It allows training models on predefined datasets, such as MS COCO, Pascal VOC, OID and KITTI, or on custom sets, with data defined in CSV files. In our case, we had to train the network on specific aerial images from the COWC dataset.

We started from the weights precomputed on the MS COCO dataset, and we did the fine-tuning for our images. For this purpose, we froze the backbone to train only the levels of the Feature Pyramid Network and the classification and regression subnetworks, without modifying the weights of the base network (Resnet-50).

The number of parameters in our network is 36,382,957, out of which only 12,821,805 are trainable, corresponding to the levels presented in Table 2. As described in Section 3.2.1, RetinaNet uses feature pyramid levels $P_{3}$ to $P_{6}$. As input, it takes the output of ResNet residual blocks, noted $C_{3}$ to $C_{5}$, according to the convention in [20].

The number of training epochs and steps was adapted according to the training approach, as described in Section 5. After each epoch, the computed weights were saved in a snapshot, so we were able to evaluate the progress of our model gradually and to restart the training from a specific point.

**Figure 4:**
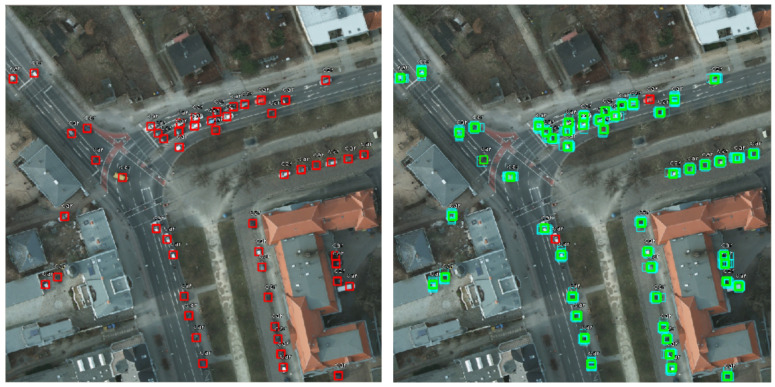
Objects considered for training (marked with green border) and ignored objects (red border). On the left-hand side, the anchors have default dimensions, while on the right-hand side they are optimized.

As previously mentioned, we optimized the anchor sizes as presented in [15]. In Figure 4, we can observe the difference between the number of cars considered for training. The situation with default dimensions for anchors is represented on the left side, and the right side contains the case with optimized anchors. The red rectangles are bounding boxes that are not considered for the training phase. So, in the default situation (left), the network will consider that our image does not have any relevant information, even if we know that there are a lot of cars in the image. After optimisation (right), the majority of rectangles are green, which means that each bounding box has a corresponding anchor box. However, as mentioned in Section 5.2.3, the optimisation did not show better results, because when we tried to perform the detection, the cars were still too small comparing to the anchors.

### 4.3. The Training Environment

The computational effort for training was high because we ran the training phase for many epochs and iterations per epoch. This involved a long training time, depending on the characteristics of the running environment. During our analysis, we used three types of machines, as described in Table 3.

Our first attempt was to train the model locally, on the personal computer, which does not allow for GPU processing. It was highly inefficient, because running an epoch with 10,000 steps on a CPU costs more than 25 h.

The improvement of the training time came with the use of Google Colaboratory. It took only three hours to run an epoch, because this time we used GPU kernels. However, it was hard to obtain significant results because the total training time was still too long.

The solution was to use an Amazon EC2 G3 Graphics Accelerated (g3s.xlarge) instance (https://aws.amazon.com/ec2/instance-types/g3/), for which a training epoch lasts about 50 min. It is also very interesting to note that the value of GPU capacity was close to 100% usage because the architecture is based on Keras and Tensorflow.

**Figure 5:**
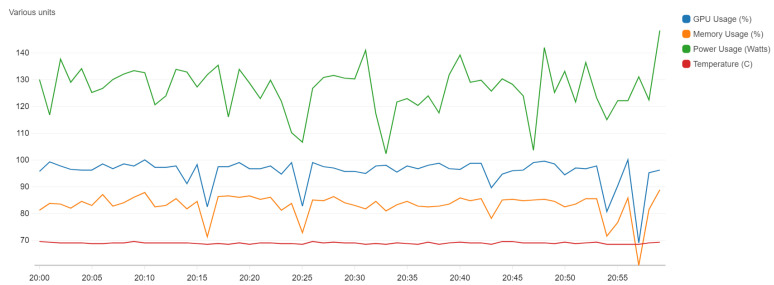
GPU monitoring for a g3s.xlarge instance used for training our model.

Figure 5 shows the resource usage on the EC2 instance, where the blue line represents GPU capacity and the orange one is the memory (with 85.8% usage). The mean power has 132 W, and the temperature is relatively constant, with a value of 70 ${}^{\circ }$C.

### 4.4. Testing

For the testing step, we applied each detection model saved in the snapshot over some new aerial images. Besides the visual result (with the detection boxes drawn around the detected cars), we also calculated the main metrics presented in Section 5.1.

The computed results depend on the values of two parameters, considered both during training and testing, namely the score threshold and the IoU threshold. The first one represents the value below which detection is not considered valid. After successive runs, we decided that the best value is 0.3. We also used Non-Maximum Suppresion (https://www.pyimagesearch.com/2014/11/17/non-maximum-suppression-object-detection-python/) to keep only the detection with the highest score for each object. The IoU (Intersection over Union) threshold defines the overlap between the bounding box and the anchor box of a vehicle.

**Figure 6:**
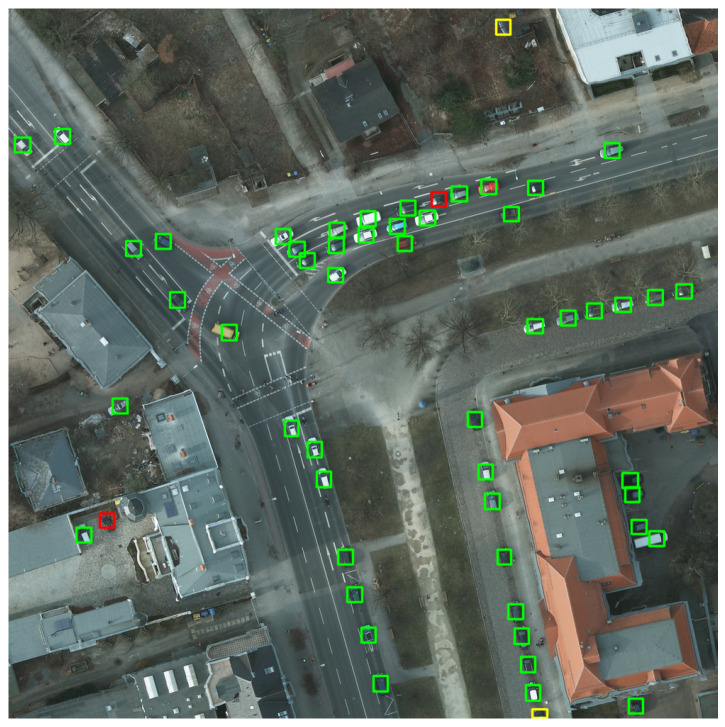
Example of detection results (green boxes are true positives, yellow boxes are false positives, red boxes are false negatives).

A sample result image is shown in Figure 6. The green boxes represent correct detected cars (true positives), the yellow ones are other objects mistaken as cars (false positives), and the red borders surround undetected cars (false negatives).

For training and evaluation, we cropped the initial images in smaller patches. However, in order to visualize the result, we rebuilt the original image, by overlapping the detection boxes and keeping the one with the highest result.

## 5. Results

The performance of our model encountered a gradual evolution while adapting the training strategy. The improvement comes by each training epoch, so to quantify it, we used the mean average precision score (mAP). This metric describes the first objective of this research, namely to identify the vehicles in static satellite images, with a high precision of detection. Regarding our second aim, to make real-time detection of cars in drone images, the performance was measured in seconds (time of detection, on each static image). This proved that our detection model works on video images as well.

### 5.1. Evaluation Metrics

For performance measurement, we used the mean average precision score (mAP), which depends on the precision and recall scores, computed as follows:

(3) $$precision=\frac{TP}{TP+FP}$$

(4) $$recall=\frac{TP}{TP+FN}.$$

In the equations above, TP is the number of true positives, FP is the false positive count, and FN is the number of false negatives recorded.

In order to compute the mAP score, we need the average precision (AP) score, defined as the area below the precision-recall graph. Based on this, the mAP score is the mean AP score for all the classes. In our situation, the only relevant class during the evaluation is the “car” class, so the mAP is equivalent to the AP, with the following formula:

(5) $$mAP=AP=\int_{0}^{1}p\left(r\right)dr.$$

**Figure 7:**
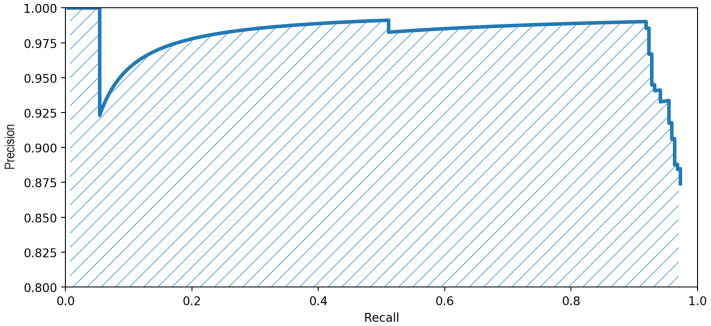
Precision-recall graph and the area below it, representing the mAP score.

Figure 7 describes the function p(r) (precision-recall). The shaded area below represents the mAP score, with a value of 0.9611 for the image of Figure 6 (more precisely, the mAP score presented here was computed for the entire overhead image whence Figure 6 was cropped.), using the best detection model obtained.

### 5.2. Approaches and Results

The first challenge in the training process was preparing the dataset to make the annotations of images compatible with the RetinaNet architecture. Moreover, a fundamental part was finding the best parameters, such that the network was trained with the appropriate configurations. Therefore, we defined five different approaches. Each one of them comes in addition to the previous one, in a gradual fashion, and shows better and more reliable results:

1. training with default parameters, considering only the “car” class (“Default”).
2. training with default parameters, considering both “car” and “non-car” classes (“Negative”).
3. anchor optimisation (“Anchor”).
4. image rescale (“Rescale”).
5. image augmentation (“Augmentation”).

The results of these approaches, expressed in mAP score, are shown in Table 4. On the first line, the model is reevaluated for the training dataset, and on the second one, we show the results for the testing set of images.

#### 5.2.1. The “Default” Approach

**Figure 8:**
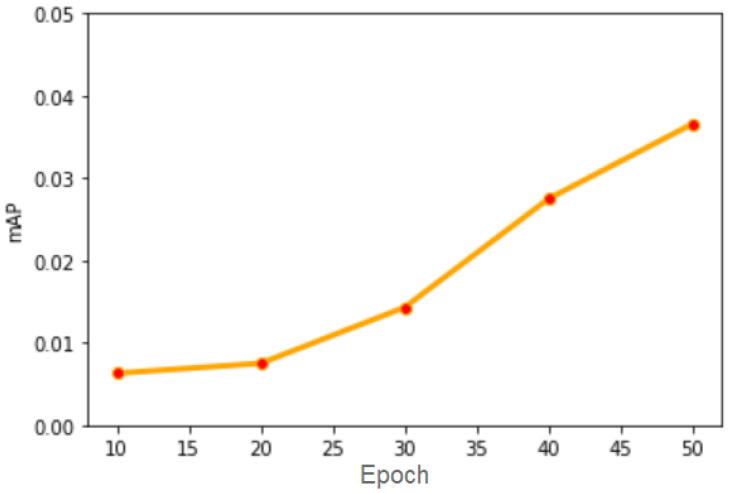
mAP score evolution with the passing of epochs for the “Default” approach.

This approach did not show satisfactory results because the training step includes only passing the images through the network, without processing them. We trained the network for 50 epochs, and we periodically evaluated the results, as illustrated in Figure 8.

However, after 50 epochs, the result still did not show great performances because the score was close to 0. After analysing the result images, we found two main shortcomings:

1. incorrect labeling of other objects in the “car” class.
2. the incapacity to detect some real cars.

#### 5.2.2. The “Negative” Approach

To solve the first problem of the previous model, we added negative examples to the training set. We annotated objects that could be easily confused with cars with the “non-car” label. The COWC dataset already had a blue dot in the middle of some relevant confounding objects, such as boats, trailers, ventilation shafts. After we computed the coordinates of their boxes, we added them into the training phase. We initialized the weights with those calculated in the previous approach and we ran 10 additional epochs.

The improvement was insignificant because, even though we managed to eliminate some false positive detections, we still had the problem of false negatives (undetected cars).

**Figure 9:**
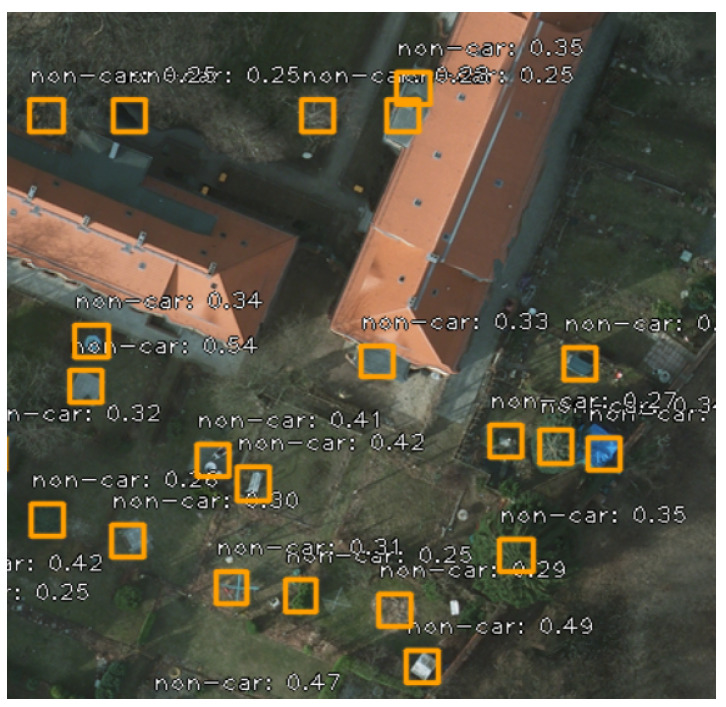
Examples of detecting non-cars.

For all the subsequent approaches, we kept the “non-car” class during training, but we only used the “car” class for evaluation. Evaluating “non-cars” is irrelevant because there are a lot of such objects, as it is visible in Figure 9, and considering them might affect the mAP score in a pointless way.

**Figure 10:**
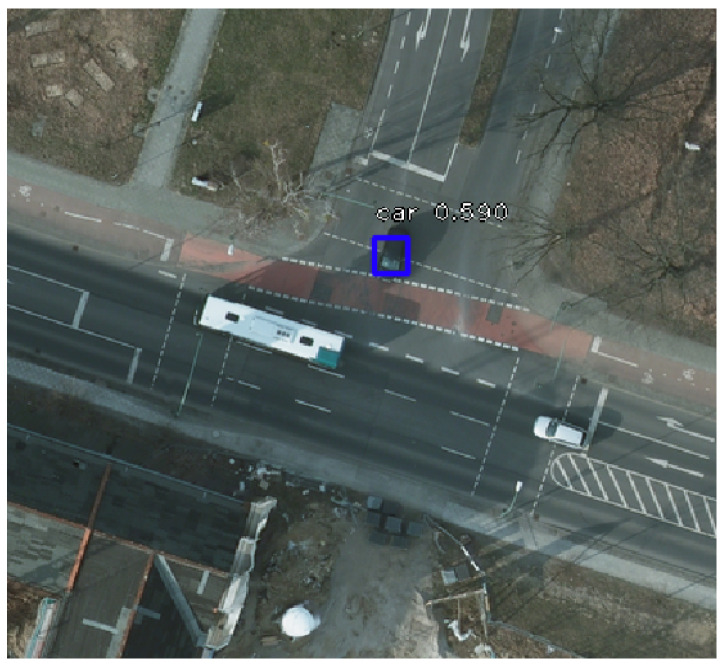
Comparison of car detection efficiency based on colour.

An additional interesting aspect to note is the deficiency in identifying white cars. The reason is their similitude with the road markings, as illustrated in Figure 10. The black car was successfully identified, while the white one was not detected. The bus was ignored as it is not included in the category of cars considered for our research.

#### 5.2.3. The “Anchor” Approach

In order to solve the problem with the incapacity to detect some cars, we optimized the anchor parameters, as described in Section 3.2.1. The improvement was insignificant, because the dimensions of the cars in images were still too small, even if the detection marks got smaller.

#### 5.2.4. The “Rescale” Approach

Analyzing the resulting images of the previous approaches, we discovered a particularity. All the original images were cropped in 1000 × 1000 px patches, with a 200 px overlap, so the bottom right margins always had smaller sizes. All the correct detections were done on these small pieces. By default, before passing through the network, all the images are resized to have the length of the smaller side equal to 800 px. This was the reason why all our 1000 × 1000 px patches went through a downscale step, which made the cars smaller and harder to detect. Only the small corner pieces had an upscale process, so the cars were clear enough to be correlated to the anchors, which had the unoptimized size of 32 × 32 px.

In this context, the “Rescale” approach comes with the solution by recalculating the length of the image side. We considered a car having 20 × 20 px, so, in order to make it correspond to a 32 × 32 px anchor in a 1000 × 1000 px image, we had to rescale the picture to 1600 × 1600 px. However, we decided to further increase the size to 2000 px, to be sure that all the training examples were correctly included.

We reset the weights before training and, after running five epochs, the results were very good, as illustrated in Table 5.

The higher values from the training set are justified by overfitting, caused by the difference in complexity between the training and testing set. The testing images contain more cars, difficult to identify both because of their density and because of their disposal.

#### 5.2.5. The “Augmentation” Approach

Starting from the previously computed weights, we made the last improvement by augmenting the images. This way, we obtained new training images by applying random transformations over the initial ones, such as rotations, translations, flips. We trained four additional epochs, and Table 6 shows the results.

**Figure 11:**
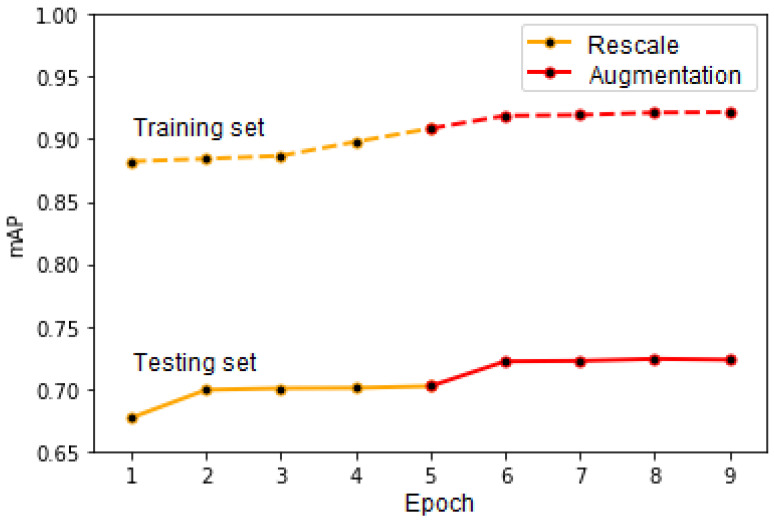
The mAP score evolution according to the approach and training epochs.

The results of the latter two approaches are illustrated in Figure 11, which presents the mAP score computed for the training set (continuous line) and testing set (dotted line). We considered that the values for the score and IoU thresholds are equal to 0.3. The first five epochs on the graph correspond to the “Rescale” approach and the last four to the “Augmentation” one. As can be observed in the figure, there still exists some overfitting, which is mainly caused by the fact that, in order to keep our network simpler, for the time being, we did not use cross-validation during its training. Furthermore, as stated previously, the testing images have larger vehicle density and are thus more complex. In the future, we wish to run our network with cross-validation enabled, which might lead to even better results.

### 5.3. Final Results

**Figure 12:**
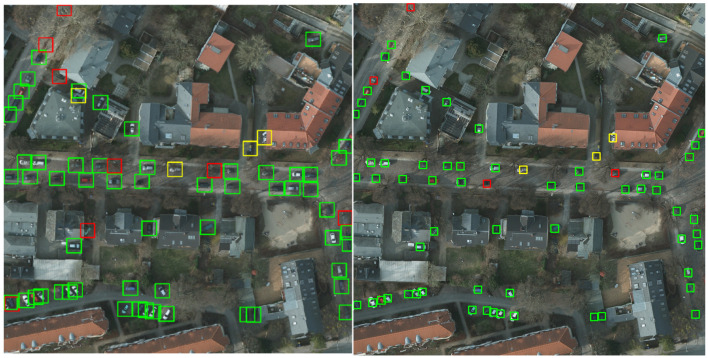
Visual comparison between the detection model obtained by the NATO Innovation Challenge winning team (left-hand side) and our detection model (right-hand side).

The best detection model is the one which takes into consideration the negative examples (“non-cars”) and combines them with the rescaling and augmenting steps during training. The image in Figure 12 is a patch extracted from a larger figure in the image splitting phase, and it shows a graphical comparison between our detection model (right-hand side) and the reference model obtained by the NATO Innovation Challenge winning team (left-hand side).

We can notice the preponderance of green rectangles that denote correct detections in both images. Yellow rectangles represent false-positive detections, where other objects have been mistaken as cars. Our model only detects three false-positive examples. One of them is a real car (so a true-positive detection) that has not been annotated in the original image. This situation might occur in multiple images, and it decreases the score, even though the detection was correctly done. In the left-side image, there is one additional false-positive example. If we consider multiple images from the test set, we can remark that the number of false-positive examples detected by our model is significantly less than the one of the NATO model. The reason is our training approach that takes into consideration the “non-car" examples. It leads to a higher precision score, obtained by our model.

The red borders show unidentified cars. The reason might be their weak exposure because they are covered by trees. In the left-side image, there are only four unidentified cars, unlike the right-side picture, where there are eight false-negative examples. It shows the higher recall value for our model.

**Figure 13:**
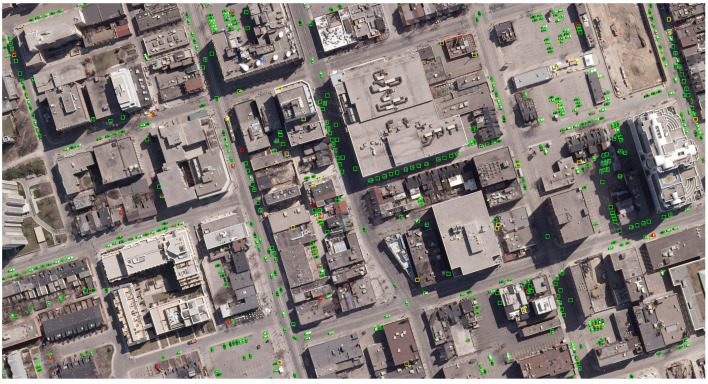
Result of car detection over an image from the testing set.

The computed F1 score, based on precision and recall, shows better results for our model (as seen in Table 7), not only for this particular patch of image in Figure 12, but also for multiple and more complex images. In their article [16], the NATO Innovation Challenge winners mention that “in more urban environments the F1-score is around 0.95”. We measured our overall model’s performance using the mAP score, but we also computed the F1 score for some images, in order to be able to make the comparison. We concluded that, in urban environments, the obtained F1 score starts from values around 0.94, and can reach 0.98 in images similar to the one in Figure 13.

We would like to highlight that the inaccurate detections are located in areas where the context is not uniform. In the parking lots and on the roads, they were correctly done. Thus, we can conclude that, when we will overlap car detection with road detection (which is something that we wish to pursue as future work), our model’s performance will considerably increase.

Starting from this premise, the problem of detecting crowded areas comes down to the ability to locate cars timely, in order to apply the model on video detection. For this purpose, we measured the time of a complete detection, and the average result shows that it takes 0.2989 s to detect all the cars in a 1000 × 1000 px image. To further highlight the capability of running our solution in a real-time fashion, we took a drone-captured overhead video and applied our network on top of it in order to detect vehicles on the fly. The experiment concluded with a live-detection video with very good results (https://drive.google.com/file/d/16Vz5Rg800Tb9V0K4QkmUw8o1L9d6snec/view?usp=sharing), which shows that our proposed solution also has potential in this direction.

## 6. Conclusions and Future Work

In this paper, we proposed and presented a model for detecting vehicles in satellite images, based on the RetinaNet architecture. We trained and tested it on the Cars Overhead With Context dataset and showed that it has a very high precision (with an mAP score as high as 0.7232) and a low detection time (around 300 ms).

However, these promising results are just the first step in developing an intelligent system for traffic fluidization. To further highlight the impact of our implementation and analyze the impact of each component of our network, we wish to extend our experimental evaluation in order to perform ablation experiments. Moreover, we wish to test our solution for various other datasets, such as DIOR [14].

For the next step, we wish to augment our current model with a street detection model that will be able to further increase the precision and reduce the detection time. One use for such a system would be to enhance existing traffic simulators such as Sim2Car [23], which can further help devise methods for reducing traffic congestion, fuel consumption, etc.

## Figures and Tables

**Table 1:** Datasets with aerial images [19].

Dataset	Image Count	Image Size (px)	Resolution (cm/px)	Car Size (px)
VEDIA	1250	512 × 512 1024 × 1024	25 12.5	10 × 20
COWC	32	2000 × 2000 19,000 × 19,000	15	24 × 48
DLR-MVDA	20	5616 × 3744	13	20 × 40
KIT AIS	241	300–1800	12.5–18	15 × 25, 20 × 40

**Table 2:** The trainable levels of the RetinaNet network. $P_{x}$ are the layers in the feature pyramid network and $C_{x}$ are the output blocks of ResNet backbone.

Level	Type	Number of Parameters	Input Level
Resnet50	Model	23,561,152 untrainable	
C5_reduced	Conv2D	524,544	res5c_relu (Resnet50)
P5_upsampled	UpsampleLike	0	C5_reduced res4f_relu(Resnet50)
C4_reduced	Conv2D	262,400	res4f_relu (Resnet50)
P4_merged	Add	0	P5_upsampled C4_reduced
P4_upsampled	UpsampleLike	0	P4_merged res3d_relu(Resnet50)
C3_reduced	Conv2D	131,328	res3d_relu (Resnet50)
P6	Conv2D	4,718,848	res5c_relu(Resnet50)
P3_merged	Add	0	P4_upsampled C3_reduced
C6_relu	Activation	0	P6
P3	Conv2D	590,080	P3_merged
P4	Conv2D	590,080	P4_merged
P5	Conv2D	590,080	C5_reduced
P7	Conv2D	590,080	C6_relu
regrssion_submodel	Model	2,443,300	P3, P4, P5, P6, P7
classification_submodel	Model	2,381,065	P3, P4, P5, P6, P7

**Table 3:** Training environment characteristics.

Running Environment	CPU Clock	CPU(s)	GPU	CUDA Kernels	GPU Memory
**Local**	1.8 GHz	8	-	-	-
**Google Colab**	2.0 GHz	2	Tesla T4	2496	16 GB
**g3s.xlarge**	2.3 GHz	4	Tesla M60	2048	8 GB

**Table 4:** The mAP score for each training approach.

	Default	Negative	Anchor	Rescale	Augmentation
**Training set**	0.0366	0.0394	0.0421	0.8896	0.9217
**Testing set**	0.0074	0.0094	0.0081	0.7020	0.7232

**Table 5:** The mAP score of the “Rescaling” approach, depending on the training epoch.

	Epoch 1	Epoch 2	Epoch 3	Epoch 4	Epoch 5
**Training set**	0.8822	0.8843	0.8867	0.8979	0.9086
**Testing set**	0.6767	0.6991	0.7003	0.7006	0.7020

**Table 6:** The mAP score of the “Augmentation” approach, depending on the training epoch.

	Epoch 6	Epoch 7	Epoch 8	Epoch 9
**Training set**	0.9086	0.9188	0.9194	0.9217
**Testing set**	0.7219	0.7223	0.7228	0.7232

**Table 7:** Precision, recall and F1-score, computed for the image patches in Figure 12.

	NATO Innovation Challenge Model	Our Model
Precision	0.9344	0.9523
Recall	0.8769	0.9375
F1 Score	0.9047	0.9448
